# Simulating Hunting Effects on the Wild Boar Population and African Swine Fever Expansion Using Agent-Based Modeling

**DOI:** 10.3390/ani13020298

**Published:** 2023-01-14

**Authors:** Chanwoo Ko, Wonhee Cho, Byungmook Hwang, Byungwoo Chang, Wanmo Kang, Dongwook W. Ko

**Affiliations:** 1 Department of Forest Resources, Kookmin University, 77 Jeongneung-ro, Seongbuk-gu, Seoul 02707, Republic of Korea; 2 Industry Academic Cooperation Foundation, Kookmin University, 77 Jeongneung-ro, Seongbuk-gu, Seoul 02707, Republic of Korea; 3 Department of Forest, Environment, and Systems, Kookmin University, 77 Jeongneung-ro, Seongbuk-gu, Seoul 02707, Republic of Korea

**Keywords:** ASF, ABM, hunting, wild boars, control strategies, South Korea

## Abstract

**Simple Summary:**

African swine fever (ASF) has caused significant damage to wildlife and domestic pig production. Since the first confirmed case in South Korea, the number of infected wild boars has continued to increase despite various management operations. Hence, this study developed the ASF expansion model based on an agent-based modeling approach to simulate management strategies for assessing the effective control of ASF. In our model, the agents’ (wild boars) behavior and daily movement range based on their ecological and behavioral characteristics by applying annual hunting scenarios from the past three years (2019.09–2022.08). Our results represented that the higher the hunting intensity, the smaller the ASF expansion area (24,987 km^2^ at 0% annual hunting rate; 3533 km^2^ at 70%). Furthermore, the complete removal of agents during the simulation period was shown to be possible through the annual hunting rate above 70%. In conclusion, an annual hunting intensity of 70% is needed to control ASF effectively.

**Abstract:**

African swine fever (ASF) is a viral hemorrhagic fever fatal to animals of the Suidae family. It has spread from Africa to Europe and Asia, causing significant damage to wildlife and domesticated pig production. Since the first confirmed case in South Korea in September 2019, the number of infected wild boars has continued to increase, despite quarantine fences and hunting operations. Hence, new strategies are needed for the effective control of ASF. We developed an agent-based model (ABM) to estimate the ASF expansion area and the efficacy of infection control strategies. In addition, we simulated the agents’ (wild boars) behavior and daily movement range based on their ecological and behavioral characteristics, by applying annual hunting scenarios from past three years (2019.09–2022.08). The results of the simulation based on the annual changes in the number of infected agents and the ASF expansion area showed that the higher the hunting intensity, the smaller the expansion area (24,987 km^2^ at 0% vs. 3533 km^2^ at 70%); a hunting intensity exceeding 70% minimally affected the expansion area. A complete removal of agents during the simulation period was shown to be possible. In conclusion, an annual hunting intensity of 70% should be maintained to effectively control ASF.

## 1. Introduction

African swine fever (ASF) is a viral hemorrhagic fever fatally affecting animals of the Suidae family. It causes continuous bleeding in the lymph nodes and intestines of the infected individuals, leading to death within 6–13 days [1,2]. Despite the high mortality rate of ASF, the ASF virus (ASFV) has high environmental resistance and can survive for a long time in the carcasses of deceased individuals, where it can be transmitted to uninfected individuals [3]. Since its emergence in Africa, ASF has spread in the African and Eurasian continents including Europe, China, Vietnam, India, Indonesia, North Korea, and South Korea. ASF is transmitted through contact between non-infected and infected individuals or their carcasses, causing continuous damage to pig farms [4,5,6].

In South Korea, the first ASF-infected wild boar individual was discovered in Yeoncheon, Gyeonggi-do in October 2019 [4]. Subsequent cases were found in Gangwon-do and Chungcheongnam-do and Chungchoengbuk-do [4,7]. Strategies for controlling ASF expansion include using natural topography (e.g., water systems and cliffs), quarantine fences to block wild boar movement, and hunting to control the population size [8]. Despite these efforts, the ASF expansion area and the number of infected individuals continues to grow [7,9,10]. 

The expansion of ASF is closely associated with the ecological characteristics of wild boars, such as core area, dietary habits, and predator status [11]. Wild boars in South Korea are reported to have an average core area of 1.18 km^2^, which varies according to sex, lifecycle, and habitat characteristics [8,12]. Adult female wild boars go through a gestation period of about three months following the breeding season (November–January), give birth to a litter, and live in groups during the rearing period of one year, during which their core area is reduced to 0.23 km^2^ [13]. Wild boars use a variety of habitats to their preference, such as avoiding threats and food abundance. They mainly prefer forested areas where they can find roots and earthworms. Furthermore, forested areas are far from human society [14,15]. They also can access farmland, grassland around the forest, or water bodies [16]. Wild boars give birth to a litter of 3–8 offspring; due to a lack of predators, wild boar populations grow quickly and a high population density is maintained, thereby enhancing the fatal ASF expansion [17,18,19,20,21,22]. 

The National Natural Environment Survey is the main source of information on the geographical distribution profiles of wild boars in South Korea; wild boar trace surveys and monitoring results are presented every 5 years [23,24]. However, this 5-year renewal cycle has limitations in predicting the spatial distribution and setting up the coping strategies for rapidly spreading infectious diseases such as ASF [25,26,27]. Therefore, to enable immediate and effective strategies for ASF expansion control, a model-based approach is needed, wherein wild boars’ ecological characteristics (e.g., habitat preference and movement pattern) can be revealed [28,29,30].

Many of the previous studies on ASF distribution and expansion profiles using a modeling approach have focused on the habitat preference of wild boars. In Europe and Asia, machine learning and regression analysis techniques have been used to predict wild boar distribution profiles, based on the relationship between wild boar abundance data and environmental factors (e.g., topography, land cover, and climate), to estimate the ASF risk areas [31,32]. Also, Cadenas-Fernández et al. (2022) estimated the wild boar habitat suitability map based on the Quality of Available Habitat map concept developed by Bosch et al. (2016) [11,33]. However, wild boar distribution prediction based on habitat preference has limited predictive power for ASF expansion and insufficient explanatory power for coping strategies because it does not reflect the process of infection expansion such as individual mobility, contact between individuals, ASF infection process, reproduction, and mortality).

More recently, an agent-based model (ABM) was developed and used to predict the infectious disease transmission process by simulating the behavioral and ecological processes regarding the hosts’ lifecycle and movement direction and distance [25,34,35]. Gervasi and Guberti (2022) [36] and Ko et al. (2021) [35] used the ABM approach to predict changes in wild boar population and ASF expansions by simulating the agents’ mobility, foraging, and breeding activities and contacts among the agents. However, both studies have limitations: Gervasi and Guberti (2022) [36] simulated in a virtual space and could not reflect environmental characteristics such as topography and climate, whereas Ko et al. (2021) [35] simulated the ASF expansion in a specific area in South Korea, therefore, the simulation results cannot be generalized to the entire country.

To address these limitations, we applied ABM to simulate ASF expansion across South Korea based on the ecological characteristics of wild boars and used it to predict ASF expansion as a result of the removal of wild boar sounders by hunting. For the ASF expansion simulation, ABM was developed using Netlogo (version 6.2.2), and the sounder control effect of hunting was assessed by analyzing the changes in the number of infected sounders according to hunting intensity. We conducted simulations for three consecutive years to (1) determine the time-series trend in the number of ASF-infected sounders, (2) estimate the change in the ASF expansion area, and (3) evaluate the effect of sounder size control according to the model-based hunting intensity on ASF expansion control.

## 2. Materials and Methods

### 2.1. Study Area

**Figure 1:**
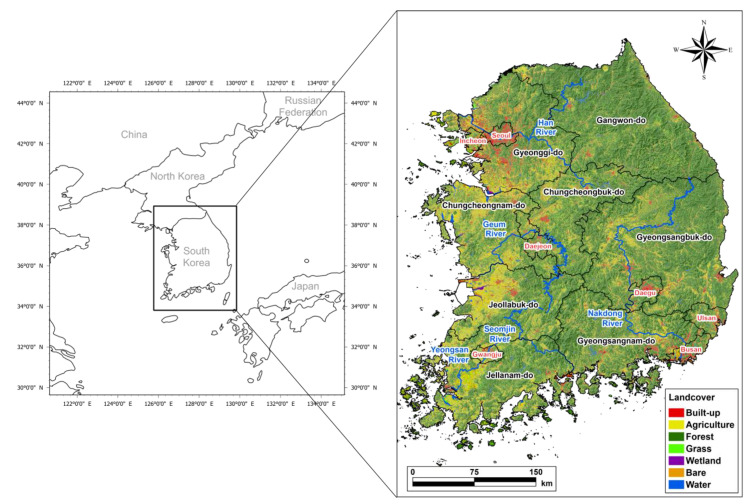
Location and land cover of the study area based on Ministry of Environment, South Korea, 2019.

The study area was set as the entire South Korean territory except for the islands (125.8°–129.63° E; 34.04°–38.61° N) to conduct ASF expansion simulation at the national level and evaluate the effect of hunting-based ASF expansion control (Figure 1). The study area measures 93,442 km^2^ (340 km in the east–west direction and 480 km in the north–south direction) and consists mainly of forest land (60%), followed by farmland (18%) and urbanized area (7%). As the major rivers, the Namhangang (South Han River), Bukhangang (North Han River), and Hangang (Han River) flow in the east–west direction across Seoul, Gyeonggi-do, and Gangwon-do (the northern part of the study area). The Geumgang flows across Chungcheongbuk-do and Chungcheongnam-do (central part), the Nakdonggang crosses Gyeongsangbuk-do and Gyeongsangnam-do (southeastern part) in the north–south direction, and the Yeongsangang crosses Jeollabuk-do and Jeollanam-do (southwestern part) in the north–south direction. The study area has a mid-latitude temperate climate with four distinct seasons: Spring and fall are mild and dry under slow migratory anticyclones, summer is characterized by high temperatures and high humidity under the influence of the North Pacific High, and winter is cold and dry due to the continental high pressure. The average minimum and maximum temperatures in August (summer) are 19.7 °C and 26.7 °C separately, and in January (winter) are 6.9 and 3.6°C. The annual precipitation is 1306.3 mm, with more than half (54%) concentrated in summer [37].

### 2.2. Model Description

**Figure 2:**
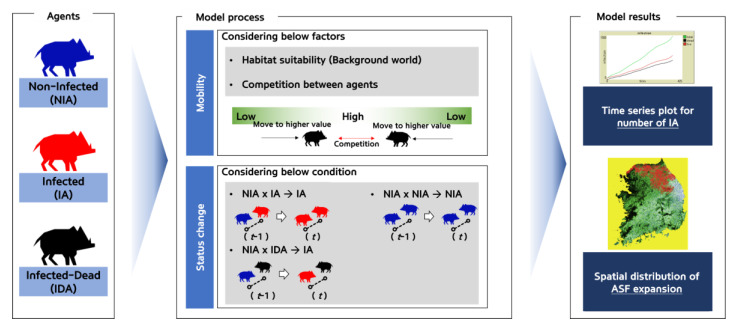
The model was designed to estimate ASF expansion based on agent mobility and status change (ASF infection) process.

In ABM, an “agent” is an object that reflects the characteristics of acting independently, by judging the given situation in each time unit defined in an autonomous and object-oriented manner, according to the rules defined by the researcher [38]. Here, the agent represents the wild boar sounder. In the ASF expansion model developed in this study, the agent’s lifecycle (foraging, breeding, death, etc.) and mobility characteristics (daily movement distance and direction) were adjusted according to the habitat preference map corresponding to the background world. Using this process, the expansion of ASF was simulated based on the contacts among the agents. The proposed model was also designed to reflect the scenario of the annual reduction rate of the agents due to hunting, which was used to assess the ASF expansion control effect (Figure 2). The agent unit was defined at the sounder level, which reflects the characteristics of the wild boar’s herd life. A sounder gauge the competition for food resources and territory according to their habitat preference at their location by a simulation time unit (referred as a “tick”), and moves to the next optimal place, during which infections occur if physical contact between the ASF-infected (both live or dead) and non-infected sounders occur. Changes in the number of wild boar sounders and the ASF expansion area were configured to be traced according to the hunting-based removal scenario. The simulation was conducted for three years with a daily tick (Figure 2).

#### 2.2.1. Background World

The background world represents a simulation area of ASF expansion ABM, which corresponds to the spatial information reflecting the habitat preference of wild boars. A habitat preference map was created using MaxEnt, a widely used species distribution model (SDM) [39]; we used 1081 wild boar data points collected through the 4th National Natural Environment Survey (2014–2018) and the topographical and climatic variables closely associated with wild boar ecology [40,41]. Elevation, slope, forest type, distance to forest, distance to road, and distance to the river were set as the topographical variables, and precipitation of the coldest quarter, temperature seasonality, isothermality, precipitation of the warmest quarter, and mean temperature of the wettest quarter were set as the climatic variables (Figure S1; Table S1, Supplementary Materials). All variables were resampled to a 1 km resolution of a raster with a resolution of a 1 km grid, and the Pearson’s correlation coefficient between each pair of variables was <0.75. The habitat preference map of wild boars produced using the MaxEnt bootstrap technique had an explanatory power of Test AUC (area under the ROC curve) as 0.72 (Figure S2, Supplementary Materials). Among the variables used, the highest contribution was made by elevation (25.3%,) followed by precipitation of coldest quarter (16.8%) and forest type at (16.7%). 

#### 2.2.2. The Agents’ Status and the Status Conversion Process

The agents of this model were simulated at the sounder level to reflect wild boar’s herd-life characteristics, and each agent had its own energy according to its foraging and breeding behavior. The sounders were classified into infected agent groups (IA), infected-dead agent groups (IDA), and non-infected agent groups (NIA) depending on their ASF infection and survival status After infection, IA moves across the background world and NIA is transformed into IA when they come into contact along the way. Although IDA cannot move, the NIA that comes across IDA is transformed into IA. The residual period of IDA was set to ≤100 days in the lower-temperature months (October–February) and up to ≤25 days in the higher-temperature months (March–September), considering the seasonal variability in the decomposition rate of the carcasses. Considering wild boars’ foraging behavior on carcasses, the residual period was reduced by 50 days once NIA encountered IDA.

#### 2.2.3. The Lifecycle of Agents

In the model, agents’ energy levels range from 0–1000 depending on the nutritional status of each sounder. The energy level changes according to the agent’s activity, and in the initial state of the model, energy scores were randomly assigned between 500 and 600. The change in the energy level is affected by the habitat preference value of the raster cell in the background map and the breeding status. Using the habitat preference index of 0.23 proposed by Ko et al. (2021) [35] as the reference value, the energy level was configured to increase by 50 for the agents staying in a place with an index ≥0.23 at each tick and reduced by 100 for the agents staying in a place with an index <0.23. The breeding-related energy change was configured to create a new sounder with the same energy using half of the energy possessed by a random sounder in March and April, which corresponds to the rearing period of young in the simulation period. Any sounder is allowed to reproduce only once over one breeding period.

An agent’s death is determined by its level of energy, lifespan, and infection status. A NIA with zero energy during the simulation period disappears from the background world. The maximum lifespan was set to 15 years, and when the age, which is randomly assigned between 0 and 15 years at the initial state of the model, reaches 15 years, the agent is considered dead.

#### 2.2.4. Agents’ Mobility and ASF

NIA and IA were moved to a location with a higher habitat preference index among eight cells adjacent to the current location. In addition, to reflect the competition between sounders, a weaker sounder moves away when an agent with higher energy level exists within an activity radius of 1 km. In this process, when NIA and IA encounter each other, NIA is infected and transformed into IA (Figure 2).

#### 2.2.5. Agent Culling Process

Before running the model, the annual ASF control intensity is set, and a certain proportion of sounders is configured to disappear according to the hunting-based control intensity at every tick. The same hunting intensity was applied to both IA and NIA, and the population reduction according to the set intensity was calculated with the following equation [36]

(1) $$H_{d}=1-\sqrt[{365}]{(1-H_{0}})$$

where *H_d_* represents the value determining the number of sounders removed at each tick, which decreases in proportion to the annual hunting intensity *H_0_*. Hunting target agents are selected from IA and NIA as those to whom a value lower than *H_d_* is assigned among the randomly assigned value between 0 and 1 at each tick. IA and NIA hunted down disappear from the background world according to the culling process for removing the captured wild boars. Therefore, the IA hunted down cannot contribute to ASF expansion in the background world as IDA. Here, 10 different hunting-based infection control scenarios were applied by increasing the hunting intensity from 0 to 90% at 10% intervals to evaluate the effect of IA and NIA population adjustment based on the hunting intensity on the expansion of ASF. 

### 2.3. Model Initialization

**Figure 3:**
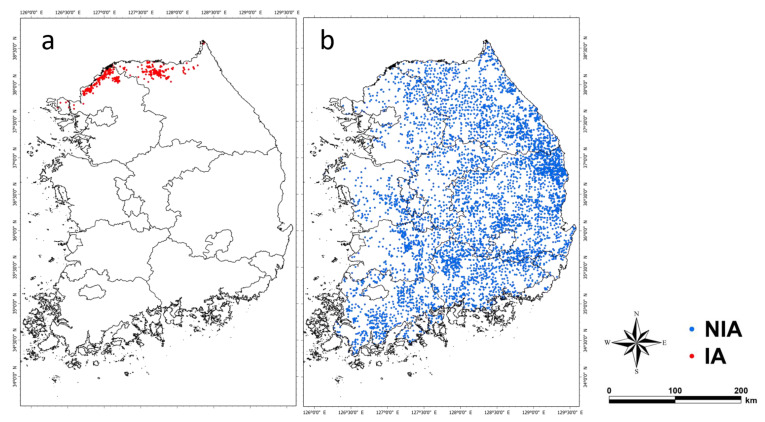
The agents (IA and NIA) generated locations in the model initialization. (**a**) The red points (656) are IA-generated locations representing where ASF-infected wild boar was observed. (**b**) The blue points (5721) are NIA-generated locations representing where wild boar or wild boar signs were observed.

NIA and IA were generated in the initial background world to assess the efficacy of hunting-based ASF expansion control. NIA was generated at 5721 points where wild boar traces were found in the 2nd, 3rd, and 4th National Natural Environment Surveys conducted by the Ministry of Environment from 1997–2019. IA was generated at 656 points where infected individuals were found as collected by the Ministry of Environment for one year after the detection of the first infected individual (Figure 3). The simulation was set to start on 1 September 2020, and end after three years, or when all infected groups (IA, IDA) disappear during the simulation period.

### 2.4. Model Simulation and Validation

In this study, the expansion of ASF and the hunting-based ASF expansion control effect was simulated for 100 replications for 10 hunting intensity scenarios, considering the stochastic characteristics of ASF expansion as a result of the agents’ independent judgment. The infection control effect was assessed by comparative analysis of the ASF expansion area and the change in the IA population size. The ASF expansion area refers to the area where IA passed at least once in the background world at each simulation, and their mean was estimated from 100 replications. The daily number of IA was estimated by averaging the 100 replications for each hunting-based infection control scenario.

For model testing, an analysis was performed to determine the degree of concordance between the area falling within a 10 km surveillance radius from the locations of ASF-infected individuals, which were detected during the initial three months (September, October, and November 2020) by the Ministry of Environment, and the ASF expansion area estimated using the ABM established in this study. Considering the lethality of ASF, recall including false negatives was applied as the model validation index.

## 3. Results

### 3.1. Model Validation

**Figure 4:**
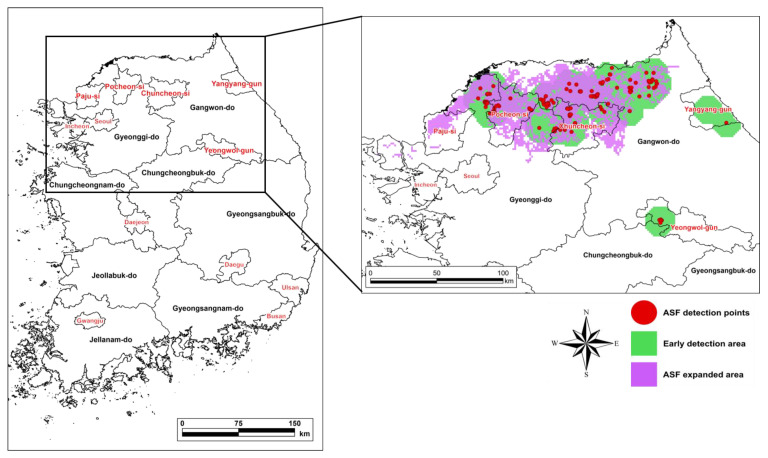
Model validation result. The validation period was September–November 2020.

The recall indicates the degree of concordance between the surveillance radius from the locations of ASF-infected individuals detected during the validation period and the corresponding ASF expansion area estimated by the model; it was calculated at 0.73 (Figure 4). ASF began to spread in the northern part of Gyeonggi-do and further to the northern part of Gangwon-do. The ASF expansion area predicted by the model coincided with the locations where the ASF-infected individuals were found (Figure 4).

### 3.2. ASF Expansion in South Korea

**Figure 5:**
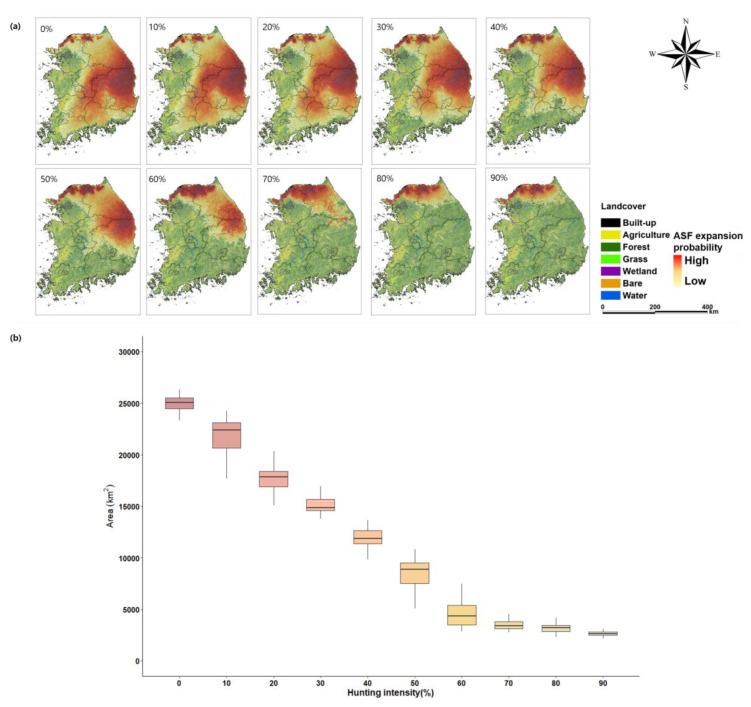
The ASF expansion area depends on the hunting rate. (**a**) Spatial distribution of the estimated ASF expansion area and (**b**) the average of ASF expansion area after a 3-year simulation.

The ASF expansion area was found to decrease with an increase in hunting intensity (Figure 5). In the scenario of 0% hunting intensity, the average three-year expansion area was estimated at 24,987 km^2^, which was limited to northern Gyeonggi-do and some parts of Gangwon-do, Chungcheongbuk-do, Gyeongsang-do, Jeolla-do, and Chungcheongnam-do. The average expansion area was 15,095 km^2^ at 30% hunting intensity, 8381 km^2^ at 50%, 4694 km^2^ at 60%, 3533 km^2^ at 70%, 3137 km^2^ at 80%, and 2661 km^2^ at 90%, indicating that the expansion area significantly decreased with an increase in the hunting intensity up to 70%, after which the expansion area reduction effect was not significant.

### 3.3. Number of ASF Infected Sounders

**Figure 6:**
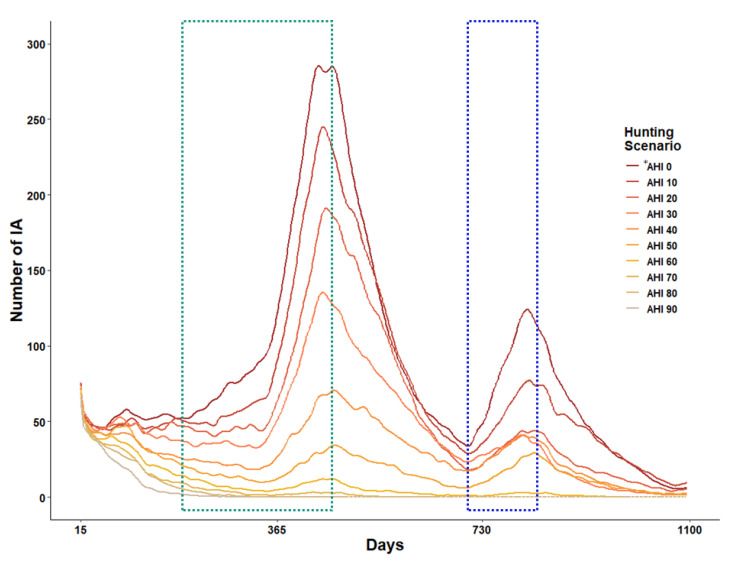
The number of IA changes in the three years simulation period depends on hunting intensity scenarios. The green and blue dash boxes represent the first and second reproduction periods of NIA. * AHI represents annual hunting intensity.

Since the IA was configured to die within 15 days of infection, the time-series number of IA decreased in the initial two weeks (Figure 6), maintained at a level of 60–70, and soared to the level of 200–350 in all scenarios during the breeding season of wild boars. The number of IA began to decrease after peaking on the 460th day when the breeding season ended, and began to increase again on the 700th day, simultaneously with the beginning of the next breeding season. During the simulation period, there were two IA-increasing periods. 

The number of IA varied according to the hunting intensity. With an increase in the hunting intensity, the rapid IA increasing rate slowed down during the two increasing periods, and both IA and IDA disappeared at a hunting intensity of 70% or higher after 500 days of simulation. However, in the 0–60% hunting intensity scenarios, IA persisted throughout three years, and the ASF expansion continued. 

## 4. Discussion

### 4.1. Model Validation

During the model validation period, the expansion of ASF by the generation and movement of IA occurred in the northern forestlands of the study area, with high habitat preference, coincided with the actual ASF expansion area, suggesting that the wild boars’ mobility characteristics were properly reflected in the model according to the habitat preference projected in the background world [42]. However, the infected individuals in the northeast and central areas of Gangwon-do, which were additionally discovered during the model validation period, were not considered in the model. This may be pointed out as a limitation of this model due to the failure to reflect the impact of the newly imported infected cases detected during the simulation period and the cases undiscovered during the early ASF expansion period [42]. It may also be attributable to the changes in wild boars’ home range due to disturbances caused by hunting operations undertaken to prevent the further expansion of infection [43,44]. Therefore, to set up model-based strategies to prevent ASF expansion at an early stage, it is necessary to improve the model to take into account the impact of unconfirmed infected individuals and changes in wild boars’ behavior caused by artificial disturbances. Nevertheless, during the model validation period, the recall rate was as high as 0.73, which is a sufficiently high level in assessing the three-year simulation of ASF expansion and the effect of hunting-based infection control, which were the objectives of this study. 

### 4.2. The Spatial Distribution of ASF Expansion 

The reduction rates for the ASF expansion area and the number of IA showed significant differences between <60% and ≥60% hunting intensities. The number of IA continued to decrease over time at ≥60% hunting intensity and disappeared after 500 days at ≥70% hunting intensity (Figure 6). This suggests that high-intensity hunting operations play an important role in preventing the contact between IA and NIA, which is consistent with a case study by the European Food Safety Authority (2018) [45]. These results highlight the importance of high-intensity hunting operations and the removal of dead individuals for ASF infection control in the future. 

When the hunting intensity was ≥70%, the ASF expansion area reduction rate and the number of IA decreased, suggesting that the infection control effect decreases at a hunting intensity ≥70% (Figure 5). In addition, given the fact that a surge in the number of IA appeared in tandem with an increase in NIA density after the breeding season, it is considered important to undertake hunting operations before the breeding season begins [46]. In areas where hunting is impossible due to low accessibility, installing quarantine fences may assist in blocking wild boar movement or in identifying major movement routes to plan intensive hunting operations [19,47,48]. 

The ASF expansion mainly occurred in forestlands with high habitat preference (Figure 1 and Figure 5a). IA expanded to the forest areas of Gangwon-do at all hunting intensities, and further to northern Jeollabuk-do and southern Gyeongsangbuk-do across the Taebaek Mountains at ≤50% hunting intensity. Conversely, IA did not expand in southern Gyeonggi-do and Chungcheongnam-do during all hunting intensities. This reflects the low mobility of IA and NIA in the Seoul Capital Area with low habit preference and the Namhan River water system, thus blocking ASF expansion. Likewise, farmlands near the Nakdong River and built-up areas such as the greater Daegu area hindered the movement of IA and NIA, indicating that the expansion patterns were divided into Jeollabuk-do and Gyeongsangbuk-do at a hunting intensity of ≤50%. This suggests that it is necessary to take advantage of the high- and low-preference regions for wild boars’ habitat and movement to enhance the efficacy of ASF control strategies [19]. 

The ASF expansion area decreased with an increase in the hunting intensity, especially markedly up to 70% (Figure 5b). At 70% hunting intensity or higher, the effect of expansion area reduction was not significant, presumably because the IA and NIA populations were sufficiently reduced at 70% of the hunting intensity such that the infected individuals did not reach the southeastern part of Gangwon-do because (Figure 3 and Figure 5a).

The number of IA varied depending on the hunting intensity. While the IA population increased rapidly from the 200th day onwards at a 0% hunting intensity, it increased only partially after the 350th day at a 70% hunting intensity (Figure 6). That is, the higher the hunting intensity, the smaller the number of IA and the greater the scope of delay in expansion. However, the number of IA remained stable at a hunting intensity of ≥80%. These results are expected to serve as a useful basis for setting up infection control strategies for infection prevention, delay, and control appropriate for the ASF expansion control goal, and for estimating the human and material resources and costs required for hunting operations [49,50].

## 5. Conclusions

In this study, we developed a model that reflects the ecological characteristics of wild boars and the ASF infection process to evaluate the efficacy of hunting-based ASF infection control. The ABM developed for this purpose reflected the ASF expansion process considering the interactions between the agents (wild boars) and the background world (environmental characteristics of the study area). Furthermore, by allowing the number of sounders to be adjusted according to the hunting intensity as configured by the researcher, the ASF expansion candidate areas and the potential number of infected agents (IA) can be estimated based on the hunting intensity scenarios. Therefore, the proposed model is expected to be highly useful for setting up ASF infection control strategies.

The simulations performed in this study led to the findings that ASF spreads mainly in areas with high habitat preference and that ASF expansion can be limited at a hunting intensity of ≥70%. The number of ASF-infected wild boar sounders increased concurrently with the increase in the number of sounders after the breeding season and during the rearing period. Moreover, the number of infected sounders converged to zero at a hunting intensity of ≥70%. Therefore, the timing of hunting operations and the selection of hunting intensity suitable for the infection control goal are very important for ASF expansion prevention.

## Data Availability

Not applicable.

## Supplementary Materials

The following are available online at https://www.mdpi.com/article/10.3390/ani13020298/s1, Figure S1: The spatial distribution of variables used for MaxEnt model, Figure S2: The results of wild boar habitat suitability (Background world), Table S1: The variables used for MaxEnt model.
