# Supplementary material for: Simulating Hunting Effects on the Wild Boar Population and African Swine Fever Expansion Using Agent-Based Modeling
Source: Animals (Basel). 2023 Jan 14;13(2):298. doi: 10.3390/ani13020298 (PMC9854879; doi:10.3390/ani13020298)
Supplement: Supplementary file 1 [file animals-13-00298-s001.zip › animals-2094286-supplementary.pdf]

Supplementary Figures & Tables

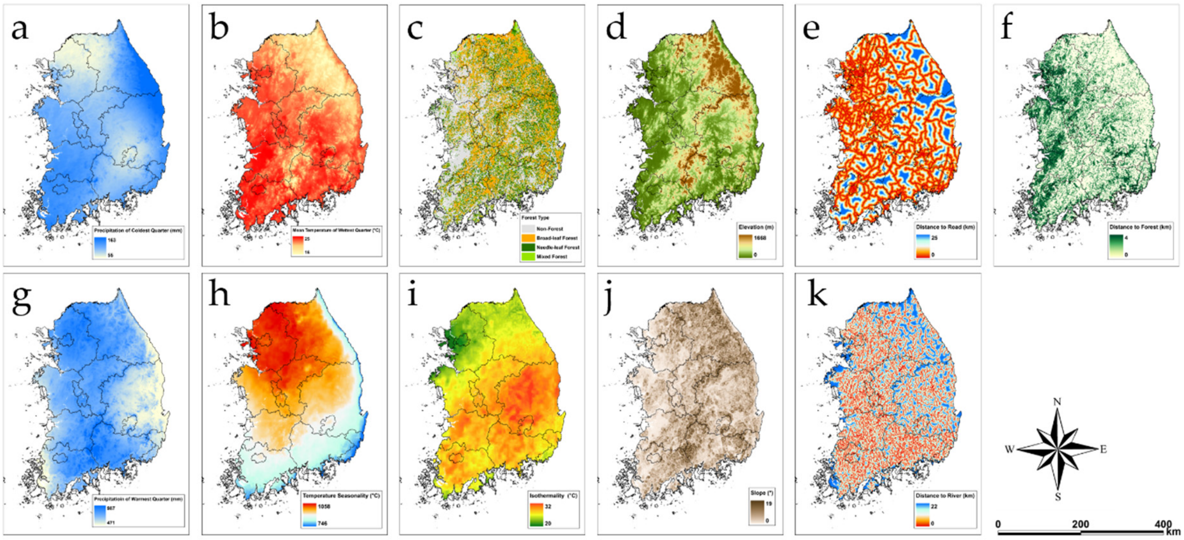

Figure S1: The spatial distribution of variables used for MaxEnt model. (a) Precipitation of Coldest Quarter (b) Mean Temperature of Wettest Quarter (c) Forest type (d) Elevation (e) Distance to road (f) Distance to forest (g) Precipitation of Warmest Quarter (h) Temperature Seasonality (i) Isothermality (j) Slope (k) Distance to river.

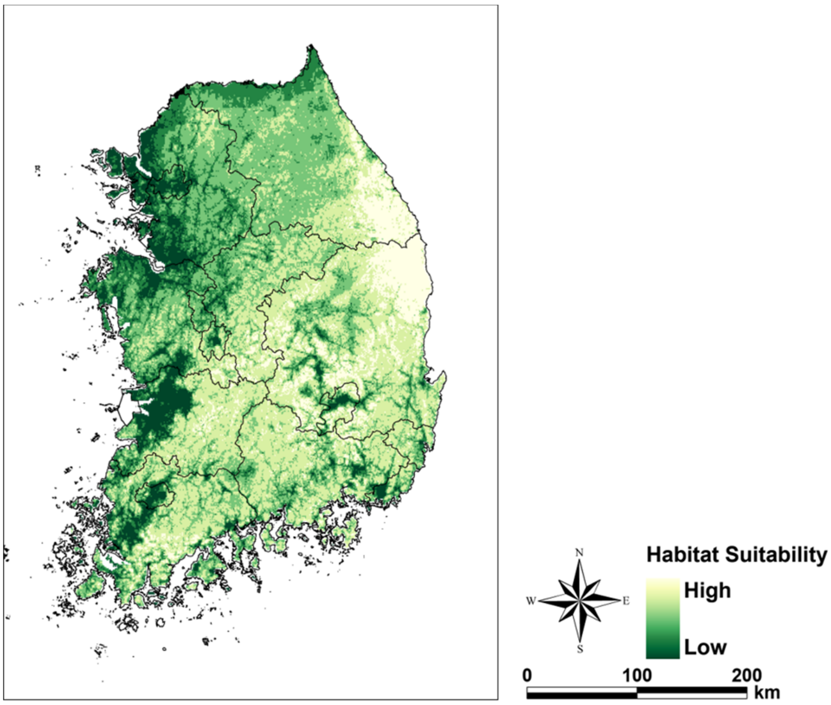

Figure S2: The results of wild boar habitat suitability (Background world).

Table S1: The variables used for MaxEnt model.

Variable	Unit	Value (Min-Max)
Elevation	m	0-2555
Precipitation of Coldest Quarter	mm	55-163
Forest type	-	Categorical
Distance to Forest	km	0-4
Temperature Seasonality	-	746-1058
Isothermality	-	20-32
Slope	°	0-19
Precipitation of Warmest Quarter	mm	471-987
Distance to road	km	0-25
Mean Temperature of Wettest Quarter	℃	16-25
Distance to River	km	0-22
